# High Residual Platelet Activity in Response to Acetylsalicylic Acid in Acute Coronary Syndrome: A New Challenge for Antiplatelet Treatment?

**DOI:** 10.5935/abc.20190199

**Published:** 2019-09

**Authors:** Dário C. Sobral Filho, José Gildo de Moura Monteiro Júnior

**Affiliations:** 1 Faculdade de Ciências Médicas - Universidade de Pernambuco, Recife, PE - Brazil; 2 Unidade Coronária do PROCAPE (Pronto Socorro Cardiológico de Pernambuco), Universidade de Pernambuco, Recife, PE - Brazil

**Keywords:** Acute Coronary Syndrome, Oxidative Stress, Plaque, Atherosclerosis, Aspirin/therapeutic use, Platelet Aggregation Inhibitors

Cardiovascular diseases (CVD) are the leading cause of death in the world, with coronary heart disease being the main etiology, accounting in 2016 for 31% of global deaths.^1^ Myocardial infarction (MI) is usually due to changes in the arterial wall or thrombotic occlusion of a coronary vessel caused by the rupture of a vulnerable plaque.^1,2^ Instability in the atherosclerotic plaque is the result of local and systemic oxidative stress, thus leading to platelet activation and formation of aggregates in the circulation.^3^ The major function of platelets is as part of the homeostatic mechanism, halting blood loss after tissue trauma, but in oxidative conditions, they are associated with various CVD such as hypertension, heart failure, stroke, diabetes and atherosclerosis.^3^

Previous studies have shown the importance of aspirin in reducing cardiovascular events in patients with coronary artery disease, hence the importance of anti-platelet aggregation in acute and chronic coronary syndromes.^4-7^ However, in this issue of the *Arquivos Brasileiros de Cardiologia*, Dracoulakis et al.^8^ demonstrate the high residual variability in response to aspirin in patients with non-ST-elevation acute coronary syndrome, comparing acute and late phases, correlating with laboratory evaluation tests of platelet aggregation and the variation of inflammatory markers (C-reactive protein and interleukin-6). In this study, the authors demonstrate statistically significant differences in response to aspirin during the acute and late phases of acute coronary disease.

Oxidative stress represents an imbalance between the production of reactive oxygen species (low density oxidized lipoproteins - oxLDLs and the catalytic subunit of NADPH oxidase - NOX2, among others) and the cellular antioxidant system (ascorbate / a-tocopherol pair, glutathione, glutathione peroxidase (GPx), heme oxygenase 1, superoxide dismutase 1 and 2 -SOD1 and SOD2, and catalase, among others), contributing to the development of atherosclerosis that eventually leads to thrombosis, the main cause of heart attacks and strokes.^1,9-12^ Reactive platelet oxygen species are mainly generated by the reduction of nicotinamide adenine dinucleotide phosphate (NADPH) oxidase.^3,9^ NOX2 is a platelet-expressed NADPH oxidase isoform and an important thrombosis regulator associated with platelet activation.^3^ Thus NOX2 has a prominent role as shown by the antiplatelet effects caused by the inhibition of NOX2 activity, resulting in impaired production of platelet, lower calcium mobilization and GPIIb/IIIa activation and usually inhibition of platelet aggregation.^9^ There is an increase in P-selectin and sCD40L plasma levels associated with increased NOX2 activity, oxLDL triggering foam cell formation and accumulation in atherosclerotic plaques, leading to platelet activation.^1^ Thus, platelets are oxidized by LDL, with activation via specific oxLDL receptors, both effects being mediated by NOX2 activation.^13^ However, there are complex enzymatic and non-enzymatic pathways involved in the formation of reactive oxygen species by cells, as demonstrated by Eduardo Fuentes et al.^1^ A Genetic deficiency of the enzyme is associated with a very rare illness (chronic granulomatous disease - CGD), which is characterized by the absence at NOX2 (X-linked CGD) or more rarely by lack of cytosolic subunits such as p47phox.^9^ This has been corroborated by the discovery of NOX2 on the platelet surface and by the demonstration, that as with leucocytes, platelet NOX2 is essential for the production reactive oxidant species. Accordingly, platelets from patients with NOX2 hereditary deficiency not only reduced F2-isoprostanes but also enhanced nitric oxide generation.^10^ Furthermore, NOX2 is important for platelet aggregation because O_2_^-^ is rapidly dismutated to H_2_O_2_.^10^ Animals treated with apocynin, which hampers p47^phox^ translocation to NOX2, disclosed reduced platelet H_2_O_2_ formation and age-related thrombosis.^10^ Studies have revelated the importance of H_2_O_2_ as a trigger of platelet activation and thrombosis, including the role of GPx, another enzyme that destroys H_2_O_2_. Animals over-expressing GPx1, platelet activation as well as platelet-related thrombosis were significantly inhibited.^10^ These data indicate that NOX2 plays a major role in platelet activation via different mechanisms: formation of F2-isoprostanes, inhibition of NO and production of H_2_O_2_.^10^ Patients with coronary atherosclerosis have a higher platelet reactivity, which may represent an increased risk of periprocedural MI. Approximately one-third of patients presenting an acute ST-segment elevation MI, even with coronary stenting, develop a “no-reflow” phenomenon that is associated with increased platelet activity or inadequate platelet inhibition at the time of MI.^1^ Therefore, oxidative stress may be associated with increased platelet aggregation due to a diminished response to antiplatelet therapy.^1^

Multiple pathways contribute to platelet activation and aggregation by reflecting, as independent signals, thromboxane A_2_ (TXA_2_)_,_ adenosine diphosphate (ADP) and activated thrombin.^13^ These represent goals in therapeutic modulation such as cyclooxygenase-1 inhibitors, P2Y12 inhibitors, protease-activated receptors (PAR) 1 inhibitors and interindividual variability in drug responses.^14^ Platelets are heterogeneous in volume and density, biological variables that determine platelet function, playing an important role in the development of intravascular thrombus. Large platelets are metabolically and enzymatically more active than small platelets, which is reflected in the increase in mean platelet volume (MPV).^15^ In the study by Hilal Bektas et al.,^16^ the MPV value above 10.4 is a predictor of severe atherosclerosis with a sensitivity of 39% and specificity of 90% (ROC curve: 0.631, 95% CI: 0.549-0.708, p = 0.003), and can be used as a predictor of cardiac risk in patients with disease coronary artery. Another pathway includes impaired biosynthesis or inactivation of NO and/or enhanced the formation of isoprostanes, which may represent a future target of antiplatelet drugs.^17^

Antiplatelet therapy is important in the prevention of MI, and despite its proven efficacy in both acute and chronic phases, there is still a high recurrence rate of ischemic events in patients with coronary artery disease.^1,17^ Aspirin resistance may be present in 5% to 75% of patients.^1^ In a systematic review, Hovens et al.^15^ demonstrated the high variability in individual response to aspirin in different populations. There are laboratory methods such as VerifyNow (VFN), total blood aggregometry (TBA) and platelet function analyzer (PFA-100) that can assess this platelet variability.^19-21^ Oxidative stress may be associated with increased aggregation due to diminished response to antiplatelet therapy.^1^ However, the reason for this high platelet variability is still unclear despite the routine use of aspirin and the relative contribution of NOX2 as a key target of different platelet activation pathways in the treatment of acute and chronic coronary disease. Specific antioxidants may, therefore, represent a new approach to limit platelet-related vascular complications due to the presence of NOX2.
